# Investigating the causal effect of Dickkopf-1 on coronary artery disease and ischemic stroke: a Mendelian randomization study

**DOI:** 10.18632/aging.205050

**Published:** 2023-09-22

**Authors:** Peng-Fei Zheng, Jing-Jing Rong, Zhao-Fen Zheng, Zheng-Yu Liu, Hong-Wei Pan, Peng Liu

**Affiliations:** 1 Cardiology Department, Hunan Provincial People’s Hospital, Furong, Changsha 410000, Hunan, China; 2 Clinical Research Center for Heart Failure in Hunan Province, Furong, Changsha 410000, Hunan, China; 3 Institute of Cardiovascular Epidemiology, Hunan Provincial People’s Hospital, Furong, Changsha 410000, Hunan, China; 4 Department of Cardiology, The Central Hospital of ShaoYang, Shaoyang 422000, Hunan, China

**Keywords:** DKK1, CAD, IS, two-sample mendelian randomization

## Abstract

Epidemiological investigations have indicated a correlation between elevated plasma levels of Dickkopf-related protein 1 (DKK1) and the presence of atherosclerosis. However, the exact causal relationship of DKK1 with the development of coronary artery disease (CAD) and ischemic stroke (IS) remains unclear. To address this gap, our study aimed to explore their causal association using a two-sample Mendelian randomization (MR) approach. We obtained summary statistics from genome-wide association studies (GWAS) meta-analyses conducted by Folkersen et al. and Nikpay et al., which included data from 21,758 individuals for DKK1 and 42,096 cases of CAD. Additionally, we obtained data from the FinnGen biobank analysis round 5, which included 10,551 cases of IS. Eight MR methods were employed to estimate causal effects and detect directional pleiotropy. Our findings demonstrated that genetic liability to DKK1 was associated with increased risks of CAD (odds ratio [OR]: 1.087; 95% confidence interval [CI]: 1.024–1.154; *P* = 0.006) and IS (OR: 1.096; 95% CI: 1.004–1.195; *P* = 0.039). These results establish a causal link between genetic liability to DKK1 and elevated risks of CAD and IS. Consequently, DKK1 may represent a promising therapeutic target for the prevention and treatment of CAD and IS.

## INTRODUCTION

Cardiovascular diseases, particularly coronary artery disease (CAD) and ischemic stroke (IS), has emerged as a significant global health threat [1, 2]. Atherosclerosis, characterized by endothelial dysfunction, inflammatory cell infiltration, cytokine production, vascular smooth muscle cell activity, and macrophage and monocyte activation, serves as a well-established pathological foundation for CAD and IS [3, 4]. Previous research has highlighted the crucial role of endothelial dysfunction, induced by endothelial cell apoptosis and pro-inflammatory pathway activation, in early atherosclerosis [5–7]. In this context, Dickkopf-1 (DKK1), a glycoprotein secreted by cells, has garnered attention due to its ability to hinder the Wnt signaling pathway by binding to the LDL receptor-related protein 5 (LRP5) receptor on the cell membrane. Consequently, DKK1 may contribute to endothelial dysfunction and plaque instability. The underlying molecular mechanisms involve the regulation of various inflammatory factors, such as interleukin-6 (IL-6), IL-1β, tumor necrosis factor α (TNF-α), and monocyte chemoattractant protein-1 (MCP-1) [8, 9]. Furthermore, an observational study involving 291 subjects demonstrated higher plasma DKK1 levels in patients with ST-segment elevation myocardial infarction (STEMI) compared to those with non-ST-segment elevation acute coronary syndrome (NSE-ACS), suggesting the potential of DKK1 plasma levels as prognostic indicators for the severity and stability of coronary atherosclerosis [10]. Similarly, another observational study led by He et al. investigated the association between DKK1 and acute IS onset in 124 stroke patients and 62 healthy controls. They found an independent association between elevated DKK1 levels and acute IS onset, thus identifying DKK1 as an independent risk factor for this condition [11]. These findings imply that DKK1 could be a potential therapeutic target for the management of CAD and IS. Nevertheless, direct evidence linking DKK1 to the risks of CAD and IS remains limited.

Given the inherent limitations of observational studies, such as confounding and reverse causality, it is crucial to employ more robust methods to investigate causal relationships between variables. Mendelian Randomization (MR) is one such method widely used to infer causality by utilizing genetic variations that are associated with the exposure of interest as instrumental variables (IVs) [12]. Since genetic variations are randomly assigned at conception, their association with outcomes is less susceptible to environmental confounding factors. To the best of our knowledge, the causal relationship between DKK1 and the risks of CAD and IS has not been established using MR. Therefore, the objective of this study was to assess the causal effect of DKK1 on the risk of CAD and IS by utilizing the MR framework.

## RESULTS

### Selection and validation of SNPs

A total of 25 independent Single Nucleotide Polymorphisms (SNPs) that exhibited a significant correlation with DKK1, with an r^2^ < 0.001 between each other, at a genome-wide significance level of *P* < 5 × 10^−8^ were identified. However, among these SNPs, rs7896518 was excluded from subsequent analysis due to its association with multiple confounding factors, such as blood lipids, blood pressure, and body mass index. Consequently, 24 SNPs were selected for further analysis, as outlined in Supplementary Table 1. Notably, all of these SNPs demonstrated *F* statistics greater than 10, indicating the absence of bias in weak instrumental variables (IVs). Moreover, a comprehensive overview of the additional characteristics of these SNPs, as well as their associations with clinical outcomes, including CAD and IS, can be found in Supplementary Table 2.

### MR estimates

**Figure 1:**
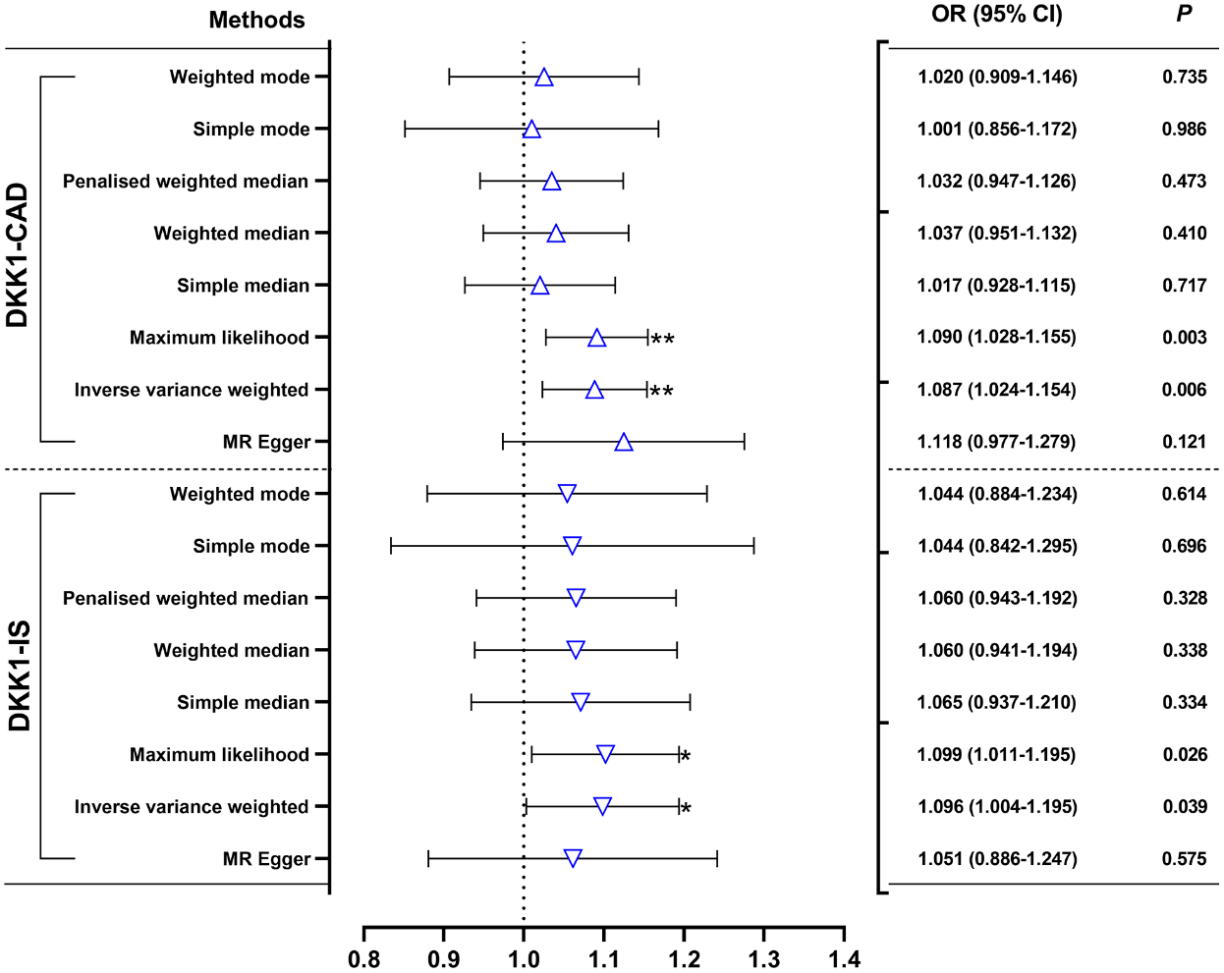
**Effects of genetically predicted DKK1 on the risks of CAD and IS.** OR, odds ratio; CI, confidence interval. *p < 0.05, **p < 0.01. DKK1, Dickkopf-related protein 1; CAD, coronary artery disease; IS, ischemic stroke.

**Figure 2:**
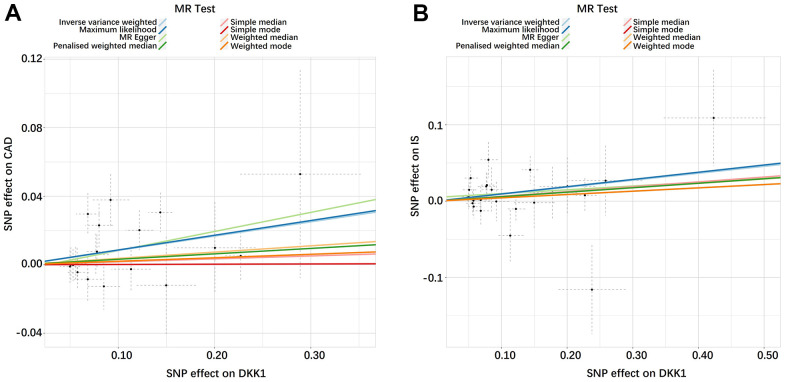
**Scatter plots of the estimated SNP effects on DKK1 (x-axis) plotted against the estimated SNPs effects on CAD and IS (y-axis).** (**A**) DKK1-CAD; (**B**) DKK1-IS. The slope of the line corresponds to a causal estimate using a different method. DKK1, Dickkopf-related protein 1; CAD, coronary artery disease; IS, ischemic stroke.

**Figure 3:**
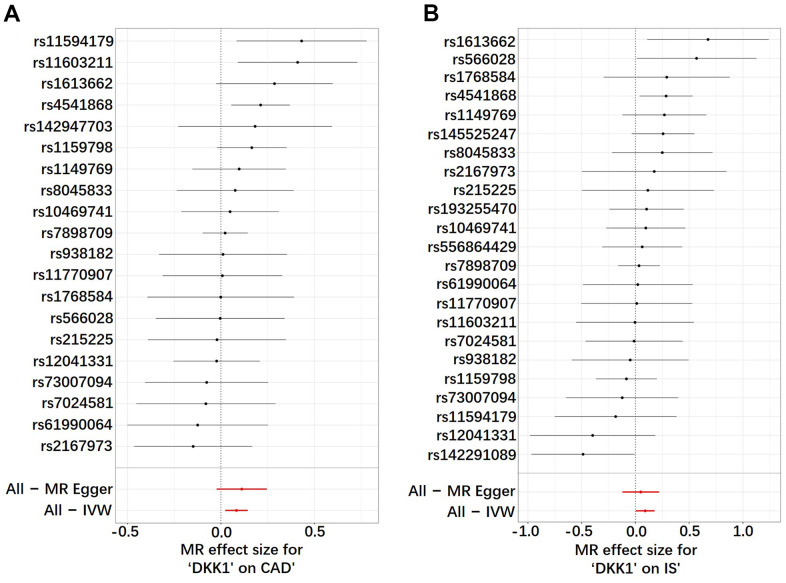
**Analysis of the single- and multi-SNP effects on the development of CAD and IS.** (**A**) DKK1-CAD; (**B**) DKK1-IS. In the forest map, each black dot represents a single SNP as instrumental variable and the red dot shows the use of IVW results for all SNPs. DKK1, Dickkopf-related protein 1; CAD, coronary artery disease; IS, ischemic stroke; SNP, single nucleotide polymorphism; IVW, inverse variance weighted.

The findings in Figure 1 and Supplementary Table 3 demonstrated that, according to the random-effects inverse-variance weighted (IVW) analysis, DKK1 was indeed a risk factor for both CAD and IS. Specifically, the analysis revealed an odds ratio (OR) of 1.087 (95% confidence interval [CI]: 1.024-1.154; *P* = 0.006) for CAD and an OR of 1.096 (95% CI: 1.004-1.195; *P* = 0.039) for IS. Similarly, the maximum likelihood analysis also supports DKK1 was a risk factor for CAD, with an OR of 1.090 (95% CI: 1.028-1.155; *P* = 0.003), as well as for IS, with an OR of 1.099 (95% CI: 1.011-1.195; *P* = 0.026). Moreover, although the results from other MR methods did not reach statistical significance, they consistently demonstrate a similar direction of effect. Additionally, the scatter plot (Figure 2) and the forest plot (Figure 3) further highlight the positive association between DKK1 levels and the risks of CAD and IS. Furthermore, no significant causal relationship was observed between CAD and IS with DKK1 levels, indicating the absence of reverse causality between DKK1 and the risk of CAD and IS (Supplementary Table 4).

### Sensitivity analyses

**Figure 4:**
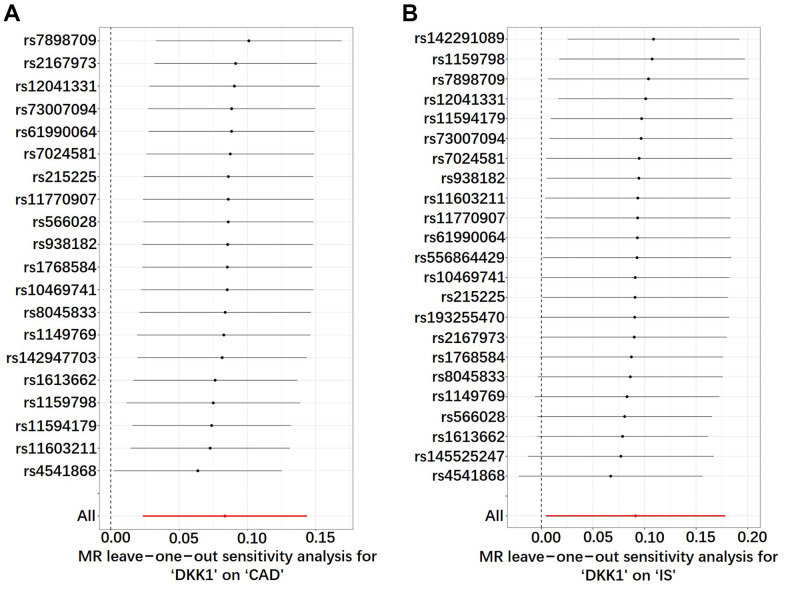
**Sensitivity analyses using the leave-one-out approach on the association of DKK1 with CAD and IS.** (**A**) DKK1-CAD; (**B**) DKK1-IS. Each black dot represents an IVW method to estimate the causal effect of the exposures on the CAD and IS. The presence of a particular SNP causing a significant change in the overall results is not excluded. DKK1, Dickkopf-related protein 1; CAD, coronary artery disease; IS, ischemic stroke; SNP, single nucleotide polymorphism; IVW, inverse variance weighted.

The leave-one-out sensitivity analysis consistently affirmed the direction and estimate of the association between elevated DKK1 levels and the risks of CAD and IS (Figure 4). Furthermore, the Two-Sample Mendelian randomization (TSMR) analysis revealed that there was no heterogeneity observed between DKK1 levels and the risks of CAD and IS, as indicated in Supplementary Table 5. Moreover, the MR-Egger intercept tests revealed no evidence of horizontal pleiotropy, with all *P*-values exceeding 0.05 (Supplementary Table 6). Additionally, no outliers were identified in the MR Pleiotropy RESidual Sum and Outlier (MR-PRESSO) analysis (Supplementary Table 7).

## DISCUSSION

Until now, the genetic associations between circulating levels of DKK1 and the risks of CAD and IS have remained unclear. To address this knowledge gap, we conducted the first MR study to clarify the genetic causalities of DKK1 levels with CAD and IS using MR methods. Given the ethical considerations and high costs associated with conducting randomized controlled trials, the MR framework provided an effective alternative for the identification of potential intervention targets and potential therapeutic strategies. Our findings indicated that every 1-standard deviation increase in DKK1 levels was associated with an 8.7% increase in the risk of CAD and a 9.6% increase in the risk of IS. Importantly, we observed no evidence of heterogeneity or horizontal pleiotropy between DKK1 levels with the risks of CAD and IS in the TSMR analysis. Furthermore, the robustness and consistency of our results were confirmed by employing eight different analytical methods. These findings established a strong and unconfounded relationship between elevated DKK1 levels and the risks of CAD and IS. Thus, DKK1 may hold significant potential as a promising molecular target for the diagnosis and treatment of CAD and IS.

Our study findings are supported by numerous pathophysiology studies. Currently, DKK1 has been recognized as a biomarker of atherosclerosis due to its significantly increased expression in atherosclerotic lesions, which contributes to endothelial activation, inflammatory responses, coronary atherosclerosis, and acute IS [13–15]. DKK1, functioning as a secretory glycoprotein, engages in competitive binding with the LRP5 receptor located on the cell membrane, impeding the Wnt signaling pathway, thereby assuming a non-lipid dependent role in vascular pathophysiology [8]. The Wnt protein family plays a crucial role in atherosclerosis, and different subtypes of Wnt proteins may have contrasting functions. For instance, Wnt5a can induce inflammation by activating the NF-kappaB (NF-κB) transcriptional pathway in vascular endothelial cells [16]. On the other hand, Wnt1 may act as an inhibitor of NF-κB activation, and compounds like geniposide and baicalin can enhance Wnt1 signaling by reducing the expression of DKK1, thereby inhibiting downstream cytokine expression, such as IL-12, by suppressing the activity of NF-κB transcription factor and subsequently slowing the progression of atherosclerotic lesions [17]. Furthermore, Ueland et al. have highlighted that DKK1 might serve as a new mediator for platelet-mediated endothelial cell activation and play a critical role in the pathological process of atherosclerosis by mediating the inhibition of the Wnt/beta-catenin signaling pathway and the activation of the NF-kB pathway [13]. These pieces of evidence underscore the crucial involvement of DKK1 in atherosclerosis, although further research is needed to elucidate its underlying mechanisms.

Our findings were consistent with several observational studies. Goliach et al. conducted a case-control study with 100 young patients with myocardial infarction and 100 healthy controls, showing that elevated DKK1 expression levels significantly increased the risk of early-onset myocardial infarction [18]. Besides, Zhu et al. conducted a clinical study with 3,178 patients with IS, demonstrating that in patients with IS for 1 year, the increased DKK1 levels were significantly associated with adverse prognosis, including all-cause mortality and severe disability [19]. In addition, Zhang et al. reported that elevated serum DKK1 levels were independently associated with an increased risk of depression three months after stroke, suggesting the potential use of DKK1 as a prognostic biomarker for post-stroke depression. Ueland et al. [20] conducted a clinical study involving 100 subjects, including 40 patients with stable angina pectoris, 40 patients with unstable angina pectoris, and 20 healthy subjects, and found that compared to healthy subjects, patients with angina pectoris had significantly increased serum DKK1 levels; meanwhile, compared to stable angina pectoris and healthy subjects, patients with unstable angina pectoris maintained higher serum DKK1 levels [13]. Moreover, an observational study with 291 subjects demonstrated higher plasma DKK1 levels in STEMI patients compared to those with non-ST-segment elevation acute coronary syndrome (NSE-ACS), suggesting the potential of DKK1 plasma levels as prognostic indicators for severity and stability of coronary atherosclerosis [10]. These findings demonstrate that DKK1 plays a crucial role in atherosclerotic diseases. Although these observational studies provide valuable insights, they cannot establish causality due to confounding factors and reverse causal bias. Therefore, the direct causal relationship between elevated DKK1 levels and the risks of CAD and IS remains uncertain. In contrast, our study utilized eight MR methods to consistently demonstrate a significant genetic correlation between plasma DKK1 levels and the risks of CAD and IS, providing evidence for a direct causal relationship between DKK1 levels and the risks of CAD and IS in the European population.

Our study indeed had some limitations that should be considered. Firstly, the effect estimates of SNPs on DKK1 levels and the risks of CAD and IS were derived from populations of European ancestry, which restricted the generalizability of our findings to other ethnic groups, and further investigations would be necessary to determine the causal relationship of DKK1 with CAD and IS in diverse populations. Secondly, although our study provided evidence for the association of DKK1 with CAD and IS, the underlying molecular mechanisms by which DKK1 contributes to these conditions are not fully understood. Additional *in vivo* and *in vitro* studies are required to elucidate the key molecular pathways involved in DKK1-mediated pathogenesis of CAD and IS.

In conclusion, our findings demonstrate a significant genetic correlation between elevated levels of circulating DKK1 and increased risks of both CAD and IS. This suggests that DKK1 might be a promising therapeutic target for the prevention and treatment of CAD and IS.

## MATERIALS AND METHODS

### Data sources

The genetic associations of the DKK1 protein were obtained from a GWAS meta-analysis conducted by Folkersen et al. [21], which analyzed 90 circulating cardiovascular proteins using data from 13 cohorts, comprising a total of 21,758 participants. The outcome data for CAD, which included 42,096 cases and 99,121 controls, were extracted from a GWAS meta-analysis by Nikpay et al. [22]. The outcome data for IS, including 10,551 cases and 202,223 controls, were obtained from the FinnGen biobank analysis round 5 (https://www.finngen.fi/). All the GWAS data utilized in our study, including DKK1 (dataset ID: ebi-a-GCST90012060), CAD (dataset ID: ebi-a-GCST003116), and IS (dataset ID: finn-b-I9_STR_EXH), are publicly available on the MRC IEU OpenGWAS data infrastructure [23] (https://gwas.mrcieu.ac.uk). It is important to note that the included GWAS datasets only pertain to European populations. Ethical approval was not required for our study as it involved a secondary analysis of publicly available data.

### Study design and selection of IVs

**Figure 5:**
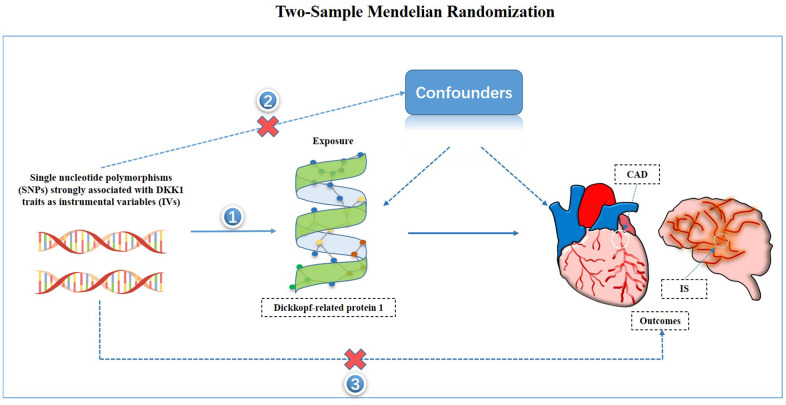
**Study hypothesis and flow chart.** CAD, coronary artery disease; IS, ischemic stroke.

The study hypothesis and flow chart are presented in Figure 5. TSMR analysis [24], a widely used method, was performed to evaluate the causal relationship between the exposures and the outcome in this study [25]. IVs were selected from SNPs that showed a significant association with DKK1 in the GWAS meta-analysis conducted by Folkersen et al. [21]. A genome-wide significance threshold of *P* < 5 × 10^−6^ [26, 27] was used for SNP selection. To ensure independence between selected SNPs, they were required to have a pairwise linkage disequilibrium (LD) r^2^ < 0.001 and located at least kb = 10000 apart from each other. The selected IVs needed to simultaneously satisfy three assumptions: (1) the genetic variants used as instrumental variables were truly predictive of DKK1; (2) the genetic variants were not associated with measured and unmeasured confounders that influence both DKK1 and CAD and IS; and (3) the genetic variants affected CAD and IS only through their effects on DKK1 and not through any alternative causal pathways. To ensure the validity of the instruments, the *F*-statistic was calculated for each SNP based on the R^2^, with a threshold of 10 [12, 28]. In cases where horizontal pleiotropy was detected in less than 50% of the instruments [29], MR-PRESSO tests were applied to remove potential outliers before each MR analysis. All IVs were extracted from GWAS using the “TwoSampleMR” package in R [30].

### TSMR analysis

In order to overcome the limitations of scarce comprehensive data within a single cohort, we adopted a TSMR approach, which allowed for analysis to be conducted in two distinct samples: one for the exposure of interest (DKK1) and another for the outcome [31]. During the TSMR analysis, four or one palindromic SNP was excluded and the remaining 20 or 23 SNPs were used to further explore the causal relationship of DKK1 with CAD and IS, respectively. In this study, eight MR methods were employed, including IVW, MR-Egger, penalized weighted median, simple mode, simple median, maximum likelihood, weighted mode, and weighted median. These methods were utilized to calculate sensitivity and obtain follow-up estimates [32, 33]. The IVW test, which is a random-effects model, was used as the primary method for estimating the causal effect values in the absence of horizontal pleiotropy to obtain unbiased estimates [12]. The other seven MR methods served as supplementary approaches to support the primary analysis. The MR-Egger method may be influenced by outlying genetic variables and provide inaccurate estimates. However, it can still provide unbiased estimates even when all selected IVs are invalid. The weighted median method, on the other hand, can provide consistent estimates of the causal effects even if up to 50% of the information in the analysis comes from invalid IVs [34]. By leveraging the strengths of each MR method, these eight approaches complement each other and provide more reliable causal effect estimates when the direction of β values is consistent. Additionally, a leave-one-out sensitivity analysis was performed to assess the reliability and stability of the causal effect estimates. The MR-Egger intercept test was used to evaluate the presence of horizontal pleiotropy, while the MR-PRESSO test was employed to detect and correct for potential outliers through outlier removal. The Cochran’s Q test was utilized to assess heterogeneity between genetic variants. Moreover, potential confounding SNPs were excluded using PhenoScanner (https://www.phenoscanner.medschl.cam.ac.uk) [35]. All these analyses were conducted using the “TwoSampleMR” R package [30].

## Supplementary Material

Supplementary Table 1

Supplementary Table 2

Supplementary Table 3

Supplementary Table 4

Supplementary Tables 5 and 6

Supplementary Table 7
