# Supplementary material for: Investigating the causal effect of Dickkopf-1 on coronary artery disease and ischemic stroke: a Mendelian randomization study
Source: Aging (Albany NY). 2023 Sep 22;15(18):9797–808. doi: 10.18632/aging.205050 (PMC10564415; doi:10.18632/aging.205050)
Supplement: Supplementary Table 1 [file aging-15-205050-s001.pdf]

SUPPLEMENTARY TABLE

Supplementary Table 1. Identification of instrumental variables.

chr.exposure	SNP	Beta	se	pval	pos.exposure	effect/otherallele	eaf
1	rs142291089	-0.2378	0.0513	0.00000351	108689300	T/C	0.0174
1	rs1768584	0.0508	0.0108	0.00000273	205240145	G/A	0.3883
1	rs12041331	-0.1133	0.0203	0.0000000236	156869714	A/G	0.0921
2	rs2167973	-0.0851	0.0168	0.000000446	33555177	C/A	0.1205
2	rs10469741	-0.2004	0.0422	0.00000208	190157500	G/A	0.0349
3	rs938182	0.0559	0.011	0.000000396	18201859	A/G	0.5913
5	rs556864429	-0.1776	0.0378	0.0000026	104057620	T/C	0.0481
6	rs566028	-0.0531	0.0113	0.00000267	147520965	C/T	0.4769
7	rs11770907	-0.0573	0.0106	0.000000064	80258630	A/G	0.5263
8	rs4541868	-0.1437	0.0124	4.05E-31	106590705	A/C	0.2471
9	rs7024581	0.1498	0.0309	0.00000127	113256645	G/A	0.0355
10	rs11594179	0.0685	0.0125	0.0000000467	104392580	T/C	0.2101
10	rs1159798	0.122	0.0142	7.65E-18	54412493	C/A	0.7656
10	rs7898709	0.2271	0.0178	1.93E-37	54423398	G/T	0.1144
10	rs1149769	0.0779	0.0113	0.0000000000067	54146097	C/G	0.6384
11	rs145525247	-0.4232	0.0788	0.0000000775	98706433	C/T	0.0097
11	rs11603211	0.0921	0.0193	0.00000173	126275830	A/G	0.0867
12	rs215225	-0.0499	0.0106	0.00000231	590545	G/A	0.4158
14	rs61990064	-0.0684	0.0146	0.00000297	92251587	G/A	0.1733
15	rs142947703	-0.2889	0.0629	0.00000433	99590146	G/A	0.0138
16	rs8045833	0.0768	0.0135	0.0000000125	88575439	A/G	0.2713
17	rs193255470	0.2586	0.056	0.00000391	56137420	T/C	0.0177
19	rs73007094	0.0578	0.0117	0.00000084	14788628	G/A	0.3779
19	rs1613662	0.0801	0.0142	0.0000000175	55536595	A/G	0.8488
