# Supplementary material for: Investigating the causal effect of Dickkopf-1 on coronary artery disease and ischemic stroke: a Mendelian randomization study
Source: Aging (Albany NY). 2023 Sep 22;15(18):9797–808. doi: 10.18632/aging.205050 (PMC10564415; doi:10.18632/aging.205050)
Supplement: Supplementary Tables 5 and 6 [file aging-15-205050-s005.pdf]

Supplementary Table 5. The results of heterogeneity analysis.

Exposures	Outcome	Method	Q	P value
DKK1	CAD	MR Egger	20.6715621723124	0.296305741630627
DKK1	CAD	Inverse variance weighted	20.9137434616642	0.341586548838615
DKK1	IS	MR Egger	24.3522979990856	0.276303645639393
DKK1	IS	Inverse variance weighted	24.7135682623749	0.310967406324591

DKK1, Dickkopf-1; CAD, coronary artery disease; IS, ischemic stroke.

Supplementary Table 6. The results of primary analysis.

Exposures	Outcome	Egger intercept	Standard error	P value
DKK1	CAD	-0.00289696585804303	0.00630846183233037	0.65157413929669
DKK1	IS	0.00493175901210229	0.00883580385579911	0.582640218062323

DKK1, Dickkopf-1; CAD, coronary artery disease; IS, ischemic stroke.
